# Boldine-Derived Alkaloids Inhibit the Activity of DNA Topoisomerase I and Growth of *Mycobacterium tuberculosis*

**DOI:** 10.3389/fmicb.2018.01659

**Published:** 2018-07-24

**Authors:** María T. García, David Carreño, José M. Tirado-Vélez, María J. Ferrándiz, Liliana Rodrigues, Begoña Gracia, Mónica Amblar, José A. Ainsa, Adela G. de la Campa

**Affiliations:** ^1^Unidad de Genética Bacteriana, Centro Nacional de Microbiología, Instituto de Salud Carlos III, Madrid, Spain; ^2^CIBER de Enfermedades Respiratorias, Madrid, Spain; ^3^Departamento de Microbiología, Medicina Preventiva y Salud Pública, Facultad de Medicina, Universidad de Zaragoza, Zaragoza, Spain; ^4^Fundación Agencia Aragonesa para la Investigación y el Desarrollo, Zaragoza, Spain; ^5^Unidad de Patología Molecular de Neumococo, Centro Nacional de Microbiología, Instituto de Salud Carlos III, Madrid, Spain; ^6^Presidencia, Consejo Superior de Investigaciones Científicas, Madrid, Spain

**Keywords:** *Mycobacterium tuberculosis*, DNA topoisomerase I inhibitor, DNA supercoiling, *N*-methyl-seconeolitsine, seconeolitsine, antituberculosis activity, drug discovery

## Abstract

The spread of multidrug-resistant isolates of *Mycobacterium tuberculosis* requires the discovery of new drugs directed to new targets. In this study, we investigated the activity of two boldine-derived alkaloids, seconeolitsine (SCN) and *N*-methyl-seconeolitsine (*N*-SCN), against *M. tuberculosis*. These compounds have been shown to target DNA topoisomerase I enzyme and inhibit growth of *Streptococcus pneumoniae*. Both SCN and *N*-SCN inhibited *M. tuberculosis* growth at 1.95–15.6 μM, depending on the strain. In *M. smegmatis* this inhibitory effect correlated with the amount of topoisomerase I in the cell, hence demonstrating that this enzyme is the target for these alkaloids in mycobacteria. The gene coding for topoisomerase I of strain H37Rv (MtbTopoI) was cloned into pQE1 plasmid of *Escherichia coli*. MtbTopoI was overexpressed with an N-terminal 6-His-tag and purified by affinity chromatography. *In vitro* inhibition of MtbTopoI activity by SCN and *N*-SCN was tested using a plasmid relaxation assay. Both SCN and *N*-SCN inhibited 50% of the enzymatic activity at 5.6 and 8.4 μM, respectively. Cleavage of single-stranded DNA was also inhibited with SCN. The effects on DNA supercoiling were also evaluated *in vivo* in plasmid-containing cultures of *M. tuberculosis*. Plasmid supercoiling densities were −0.060 in cells untreated or treated with boldine, and −0.072 in 1 × MIC *N*-SCN treated cells, respectively, indicating that the plasmid became hypernegatively supercoiled in the presence of *N*-SCN. Altogether, these results demonstrate that the *M. tuberculosis* topoisomerase I enzyme is an attractive drug target, and that SCN and *N*-SCN are promising lead compounds for drug development.

## Introduction

*Mycobacterium tuberculosis* is the causative agent of tuberculosis (TB), a major global health issue. In 2016, an estimated 6.3 million people developed TB, 1.3 million of HIV-negative people and 374,000 HIV-positive people died because of TB (World Health Organization, 2017). Nowadays, the recommended treatment for drug-susceptible TB is a 6-month regime of four first-line drugs: isoniazid, rifampicin, ethambutol, and pyrazinamide. Failures in drug supply and patients’ lack of adherence to treatment (among other factors) have resulted in the emergence of resistance to anti-TB drugs. Multidrug-resistant TB (MDR-TB) is characterized by resistance to both rifampicin and isoniazid. Treatment of these strains takes longer and requires additional drugs that are more toxic and less effective. In fact, in 2016, a total of 490,000 people developed MDR-TB globally, leading to an estimated 240,000 deaths. In addition, an estimated 6.2% of MDR-TB cases progressed into extensively drug-resistant TB (XDR-TB), defined as MDR-TB with additional resistance to a fluoroquinolone and at least one of three injectable second-line drugs (amikacin, kanamycin, or capreomycin).

Given these facts, new anti-TB drugs are urgently needed. Rifampicin, the most effective anti-TB drug, was introduced into clinical practice in the 1960s. Since then only one drug has been developed, bedaquiline, which recently was approved for MDR-TB treatment (Palomino and Martín, 2013). Even though when new drug candidates are emerging from the pipeline, and some are undergoing clinical trials (Zumla et al., 2014), investigation of new anti-TB drugs needs to continue. In order to avoid cross-resistance with already existing drugs, it is necessary to identify and characterize new targets for anti-TB drugs (Sharifi-Rad et al., 2017).

The DNA supercoiling level is an essential parameter of bacteria, given that it is a critical component of DNA replication, transcription, and recombination (Champoux, 2001). An adequate level of DNA supercoiling is maintained by DNA topoisomerase enzymes. These enzymes act on double-strand DNA, cleaving either both strands (type II enzymes) or one of the DNA strands (type I enzymes) allowing the intact segment to pass through. The cleaved DNA is then resealed before being released. DNA topoisomerase I (TopoI) has been proposed as a new antibacterial target (Tse-Dinh, 2009). Some natural compounds inhibited *in vitro* the enzymatic activity of this enzyme from *Yersinia pestis* and *Escherichia coli*, and were shown to enhance DNA cleavage (Cheng et al., 2007, 2013). Among them was a phenanthrene alkaloid able to inhibit the relaxation activity of *E. coli* TopoI, although no significant inhibition in cell growth was observed (Cheng et al., 2007). We have established TopoI as a new drug target in *Streptococcus pneumoniae* and described two novel alkaloid compounds: seconeolitsine (SCN) and *N*-methyl-seconeolitsine (*N*-SCN), derived from boldine. Both compounds inhibited *S. pneumoniae* TopoI activity *in vitro* at concentrations equivalent to those necessary to inhibit bacterial growth (∼10 μM) without affecting human cell viability (García et al., 2011). *M. tuberculosis* possess two DNA topoisomerases: one type II enzyme, DNA gyrase, which is targeted by fluoroquinolone antibiotics (Kumar et al., 2014) and one type I enzyme, topoisomerase I (MtbTopoI), which is encoded by Rv3646c (*topA*) gene. This gene has been shown to be essential for *M. tuberculosis* growth (Kumar et al., 2014).

The aim of the present study was to investigate these two boldine-derivative alkaloids as potential inhibitors of the MtbTopoI enzyme, a scarcely explored drug target. SCN and *N*-SCN inhibited growth of *M. tuberculosis* at relatively low concentrations and also inhibited MtbTopoI activity *in vitro*. In addition, their effects on DNA supercoiling were evaluated in plasmid-containing cultures of *M. tuberculosis*.

## Materials and Methods

### Bacterial Strains, Growth Conditions and Determination of Minimal Inhibitory Concentrations (MICs)

The reference laboratory strain *M. tuberculosis* H37Rv (ATCC 25618) and a panel of eight genetically distinct clinical strains of *M. tuberculosis* were used for drug susceptibility testing. This included strain GC1237, a highly transmissible strain of the Beijing lineage. A derivative of the H37Rv strain containing plasmid vector pSUM36 (Ainsa et al., 1996) was used for testing the *in vivo* effect of alkaloids on DNA supercoiling. To determine the mechanism of action of topoisomerase inhibitors, *Mycobacterium smegmatis* mc^2^155 (Snapper et al., 1990) was used along with its derivative MsPptrtopoI conditional knock-down mutant (Ahmed et al., 2015), in which levels of topoisomerase I (MsTopoI) can be reduced by addition of anhydrotetracycline (ATc). All strains were grown in Middlebrook 7H9 broth (Becton Dickinson) supplemented with 10% ADC (Becton Dickinson) and 0.05% Tween 80 (Sigma). Kanamycin (50 mg/L) was added to ensure the maintenance of plasmid pSUM36. Minimal inhibitory concentrations (MICs) were determined by microdilution as previously reported for *M. tuberculosis* (Palomino et al., 2002); MICs of drugs for *M. smegmatis* were determined by the same method except that plates were incubated for 3 days. The MIC was defined as the lowest concentration of drug that prevented change of resazurin from its oxidized form (blue) into the reduced one (pink), which is indicative of bacterial growth. Imipramine, a well-known topoisomerase-poison described previously (Godbole et al., 2015) was included as a control. For the time-kill kinetics experiments, a bacterial inoculum of 10^7^ CFU/ml was incubated in the presence of inhibitory concentrations of *N*-SCN (8 × MIC) for 24 h at 37°C; then, the culture was serially diluted in order to determine the number of viable bacteria, by plating on Middlebrook 7H10 plates; relative CFUs in comparison with untreated control cultures were determined.

### Cloning and Expression of *M. tuberculosis topA* in *Escherichia coli*

The Rv3646c (*topA*) gene from *M. tuberculosis* was amplified by PCR using 0.5 μg of chromosomal DNA from *M. tuberculosis* H37Rv strain as a template and 1 μM each of the following synthetic oligonucleotide 5′-phosphorylated primers: TopATbUP2 (5′-ATGGCTGACCCGAAAACGAAGGG-3′) and TopATbDOWN2 (5′-cgcgcgcatgcCTAGTCGCGCTTGGCTGCC-3′). The latter including a PaeI restriction site (lowercase underlined). The sequence of primer TopATbUP2 contained the ATG initiation codon and the sequence of TopATbDOWN contained the TAG stop codon of Rv3646 (both upper-case underlined). Amplification was achieved using an initial cycle of 2 min denaturation at 94°C, followed by 30 s annealing at 55°C, and a 3 min polymerase extension with Platinum Taq Polymerase high-fidelity (Invitrogen) at 68°C. This was followed by 34 cycles of 30 s at 94°C, 30 s at 55°C and 3 min at 68°C followed by slow cooling at 8°C. After completing amplification, excess oligonucleotides were removed using the QIAquick PCR Purification Kit (QIAGEN). The amplicon was digested with PaeI, cloned into plasmid vector pQE1 (TAGZyme, Qiagen) digested with PaeI + PvuII (blunt ends), resulting in recombinant plasmid pQE-MtbTopA that was introduced into *E. coli* XL1Blue. Sequencing with oligonucleotides pQE-seq3 (5′-AGCTAGCTTGGATTCTCACC-3′) and pQE-seq5 (5′-GAGGCCCTTTCGTCTTCA-3′) was performed to confirm that no mutation was introduced during cloning. The pQE1 vector/XL1 host cloning system permits the hyperproduction of 6 × His-tagged recombinant proteins encoded by genes placed under the control of a phage T5 promoter and two *lac* operator sequences. A culture of *E. coli* XL1/pQE-MtbTopA was grown at 37°C in 2 L of LB medium containing 100 μg/ml of ampicillin until it reached an OD_620_ of 0.6. Cells were induced with 1mM Isopropyl-β-D-thiogalactoside (IPTG) for 3 h, harvested by centrifugation and suspended in 50 ml of buffer A (50 mM NaH_2_PO_4_ pH 8, 300 mM NaCl, 1 mM PMSF) containing 10 mM imidazole. Triton X100 was added to a final concentration of 0.25%, and the solution was frozen at −80°C then defrosted on ice. Cells were then incubated with lysozyme (1 mg/ml) at 4°C for 60 min with lysozyme and sonicated for 100 s (5 times for 20 s, with 1 min cooling between each burst) in a Sonifier B-12 (Branson Co., Connecticut). The crude extract was centrifuged at 10.000 × *g* for 20 min and the resulting supernatant filtered using a 0.45 μm Millipore Millex^®^HA filter. The MtbTopoI protein was purified by affinity chromatography in 1 ml Ni-NTA (QIAGEN) column attached to an ÄKTA FPLC system using the standard manufacturer protocol for this column. The column was equilibrated with 10 column volumes of buffer A. Non-specifically bound proteins were removed with a 20 column volume of 10–60 mM imidazole in buffer A. Specifically bound proteins were eluted with a 40 column volume gradient of 60–350 mM imidazole. Fractions of 2 ml were collected and analyzed by SDS-10% polyacrylamide gel electrophoresis followed by Coomassie blue staining. Fractions containing a protein of the expected size, which had eluted at around 100 mM imidazole were dialyzed against buffer B (50 mM NaH_2_PO_4_ pH 8, 100 mM NaCl, 0.5 mM DTT, 50% glycerol). The purified MtbTopoI protein was stored at −20°C and thereby remained active for at least 12 months.

### Relaxation of pBR322 *in Vitro* by *M. tuberculosis* TopoI and Cleavage Assay

Reactions were carried out using 0.5 μg of supercoiled plasmid pBR322 in 200 μl of a buffer containing 50 mM Tris HCl pH 8, 20 mM KCl, 10 mM MgCl_2_, 0.5 mM DTT and 30 μg BSA/ml. After 1 h incubation at 37°C in the presence on MtbTopoI, the reaction was terminated by incubation at 37°C for 2 min after adding EDTA to a final concentration of 50 mM followed by an incubation of 1 h at 37°C in the presence of 1% SDS and 100 μg/ml proteinase K. When required, samples were ethanol precipitated and suspended in H_2_O. Reaction products were analyzed by electrophoresis in 1.2% agarose gels in 1 × TAE at 18 V for 16 h. In order to test the effect of drugs against the MtbTopoI-mediated relaxation of pBR322, the enzyme was pre-incubated with these compounds for 10 min at 4°C in a final volume of 20 μl. Electrophoresis was carried out in gels in the presence and absence of ethidium bromide (EtBr). Quantification of DNA was performed by densitometers of EtBr stained agarose gels (Quantity One, Bio-Rad Laboratories). To calculate MtbTopoI activity, the amount of the CCC form was measured and the value divided by the total amount of DNA in each well. The mean IC_50_ (*n* = 3) was defined as the concentration of drug required to achieve a 50% reduction of enzymatic activity.

For the cleavage assay, a 5′-biotin-labeled 32-mer olig-onucleotide (5′-CAGTGAGCGAGCTTCCGCTTGACATCCCAATA-3′), which contains the strong topoisomerase site of mycobacterial TopoI, was used as substrate (Godbole et al., 2015). Reaction mixtures containing 1 unit of MtbTopoI in the same buffer as above were pre-incubated with SCN for 10 min at 4°C. Then, 0.1 pmol of the DNA substrate were added and the mix was incubated for 30 min at 30°C. Reactions were terminated by adding 45% formamide dye and heating at 95°C for 5 min. Samples were resolved on 12% denaturing 7 M urea/PAGE using 1 × TBE as running buffer at 300 V. DNAs were transferred to Hybond-N^+^ membrane (Amersham) using the *Trans*-blot cell (Bio-Rad) in 1 × TAE buffer according to the manufacturer’s instructions and the blots were UV-cross-linked. Detection was performed by autoradiography using chemiluminescent nucleic acid detection module from Thermo Fisher Scientific. Enzyme activity was determined by relative quantification of band intensities with ImageLab software.

### Activity of Alkaloids on DNA Supercoiling in *M. tuberculosis* Cultures

Cultures of 50 ml of *M. tuberculosis* H37Rv containing the plasmid vector pSUM36 were grown until OD_600_ of 0.4 was reached, which corresponded to a density of 1.7 × 10^7^ bacterial cells/ml. The culture was split into three fractions, one of which was left untreated, while the other two were either treated with boldine at 500 μM (corresponding to 1 × MIC) or SCN at 15.6 μM (corresponding to 1 × MIC). After 4 h at 37°C, total DNA was extracted from half of each fraction as described previously (Ferrándiz et al., 2010). At 24 h the other half of each fraction was processed as above. DNA samples were suspended in 30 μl of distilled water and kept at −20°C until analyzed in two-dimensional agarose gels.

### Analysis of the Topology of Covalently Closed Circles

Circular DNA molecules were analyzed in neutral/neutral two-dimensional agarose gels, a technique that allows to separate DNA molecules by mass and shape. The first dimension was run at 1.5 V/cm in a 0.4% agarose (Seakem; FMC Bioproducts) gel in Tris-borate-EDTA (TBE) buffer for 17-19 h at room temperature. The second dimension was run at 7.5 V/cm in 1% agarose gel in TBE buffer for 7-9 h at 4°C. Chloroquine (Sigma) was added to the TBE buffer of both the agarose gel and the running buffer. After electrophoresis, gels were subjected to Southern hybridization. A 177-pb probe was obtained by PCR amplification of the kanamycin-resistance gene of plasmid pSUM36 (Ainsa et al., 1996) with oligonucleotides PSUM36FBIOT 5′-TCGGCAGGAGCAAGGTGAGATGA-3′ (biotinylated at 5′) and PSUM36R 5′-CCTGTCCGGTGCCCTGAATGAA-3′. This probe was used on the nylon membranes (Inmobylon NY^+^, Millipore) onto which the two-dimensional agarose gels had been transferred. Chemiluminescent detection of DNA was performed with the Phototope^®^ -Star kit (New England Biolabs). Images were captured in a VersaDoc MP400 system and analyzed with the Quantity One program (Bio-Rad).

## Results

### Inhibition of *M. tuberculosis* Growth by Alkaloids

We first tested the susceptibility of several *M. tuberculosis* isolates to boldine and its derivatives SCN and *N*-SCN (**Table 1**). In general, *M. tuberculosis* strains were more susceptible to boldine, SCN and *N*-SCN than what we reported previously for streptococci (data from García et al., 2011, are included in **Table 1** for comparison). Susceptibility to boldine was low, with MIC values ranging from 31.25 to >500 μM, in agreement with previous reports of the low anti-TB activity of this alkaloid (Guzman et al., 2010). Boldine derivatives SCN and *N*-SCN showed a much stronger antimicrobial activity, resulting in a minimum of a fourfold decrease in the MIC. For some clinical strains, such as HMS1292 (**Table 1**), the MIC of *N*-SCN was 32-times lower than that of the parent compound boldine, reaching as low as 1.95 μM, the lowest MIC we have detected. We speculated that sequence polymorphisms in the gene encoding MtbTopoI could explain the high susceptibility to alkaloids shown by some isolates, however, after sequencing Rv3646c alongside flanking regions in these strains, no polymorphisms were found. Hence, permeability, differences in the nucleoid-associated protein repertoire, or other unidentified mechanisms must be involved in basal susceptibility of mycobacteria to SCN and *N*-SCN.

**Table 1:** Susceptibilities of bacterial isolates to boldine and its alkaloid derivatives.

Bacterial isolate		MIC (μM)		
		Boldine	*N*-SCN	SCN
*S. pneumoniae*	R6	1000	16	16
*S. pneumoniae*	ATCC 6303^T^	1000	16	16
*S. mitis*	NCTC 12261^T^	1000	16	16
*M. tuberculosis*	H37Rv	>500	15.6	15.6
*M. tuberculosis*	GC 1237	31.25	7.8	7.8
*M. tuberculosis*	HMS1553	31.25	1.95	3.9
*M. tuberculosis*	HMS 1292	62.5	1.95	7.8
*M. tuberculosis*	HMS1278	>250	7.8	15.6
*M. tuberculosis*	HMS 1498	31.25	3.9	7.8
*M. tuberculosis*	HMS 1500	>250	7.8	15.6
*M. tuberculosis*	HMS 1536	>250	7.8	15.6
*M. tuberculosis*	HMS 1546	>250	7.8	7.8

### Cloning of the Gene Coding the DNA Topoisomerase I of *M. tuberculosis* H37Rv

**FIGURE 1:**
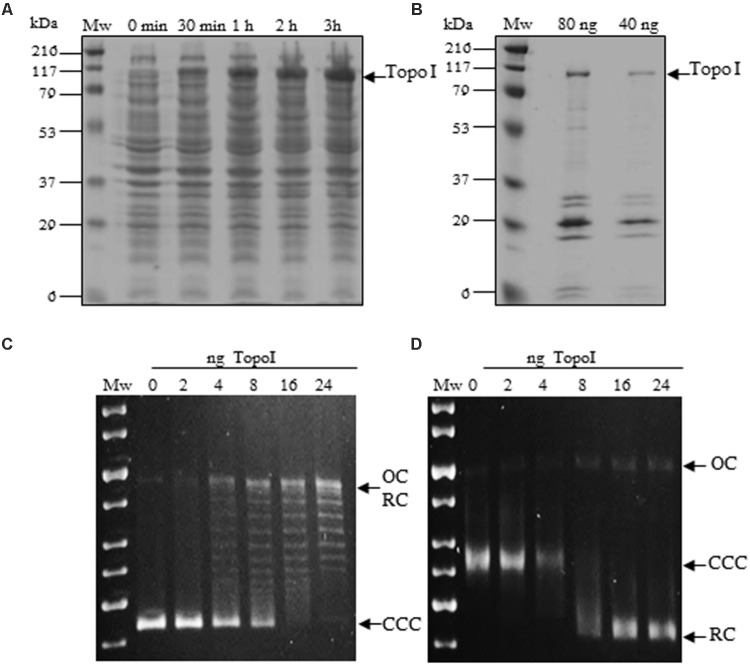
Expression, purification and activity of *Mycobacterium tuberculosis* Topoisomerase I. **(A)** Cultures of *Escherichia coli* XL1Blue containing pQE1-MtbTopA were grown in LB medium and induced with IPTG at the indicated times. Crude extract samples of 10 μl were electrophoresed in a SDS-12% polyacrylamide gel. The polypeptides were visualized by Coomassie Blue staining. **(B)** Fractions extracted from a Ni-NTA column that showed topoisomerase I activity. **(C,D)** Plasmid pBR322 (0.5 μg) was incubated with the indicated amounts of purified MtbTopoI for 1 h at 37°C. OC, relaxed open circles; CCC, covalently closed circles; RC relaxed circular plasmids forms. Mw, molecular mass standard. **(C)** Agarose gel run in the absence of EtBr. **(D)** The same samples as in C were run in the presence of 1 μg/ml of EtBr.

The MtbTopoI is encoded by *topA* gene (Rv3646c) in *M. tuberculosis* H37Rv genome. The enzyme consists of 934 amino acids and has an estimated molecular weight of 102.3 kDa (Cole et al., 1998). The *topA* gene was amplified by PCR from *M. tuberculosis* H37Rv chromosomal DNA with specific oligonucleotides and cloned into the *E. coli* plasmid pQE-1 rendering plasmid pQE-MtbTopoI, which carries the Met-(His)_6_-Gln-MtbTopoI fusion protein under the control of T5 promoter. This plasmid was introduced into *E. coli* XL1-Blue and its T5 promoter was induced by IPTG leading to overexpression of a protein with an apparent molecular weight of 100 kDa (**Figure 1A**). This is in agreement with the expected size of the fusion protein (102.3 kDa for MtbTopoI + 1.23 kDa for the N-terminal fusion). Although other proteins co-eluted with MtbTopoI (**Figure 1B**) they did not appear to interfere with the DNA relaxation activity of MtbTopoI on pBR322. The total yield of purification was 0.160 mg of MtbTopoI per 2 l culture. The final concentration was 8 mg/l and the recombinant MtbTopoI had a specific relaxation activity of 1.25 × 10^6^ units/mg (see below).

To test the DNA relaxation activity of MtbTopoI, covalently closed negatively supercoiled (CCC) pBR322 plasmid (0.86 nM) was incubated in the presence of different amounts of the purified enzyme and the reaction products analyzed in mono-dimensional agarose gels as described (García et al., 2011). MtbTopoI activity converted the CCC plasmid into topoisomers with different degrees of supercoiling that were observed as discrete bands in a gel run in the absence of EtBr (**Figure 1C**). As expected, all relaxed topoisomers (RC) migrated as a single band when the gel was run in the presence of saturating concentrations of EtBr (**Figure 1D**). A unit of MtbTopoI enzymatic activity was defined as the amount of enzyme being able to relax 50% of the CCC substrate in 1h at 37°C. This corresponded to 8 ng (3.6 nM) of purified enzyme.

### Inhibition of *M. tuberculosis* TopoI Activity by Alkaloids

**FIGURE 2:**
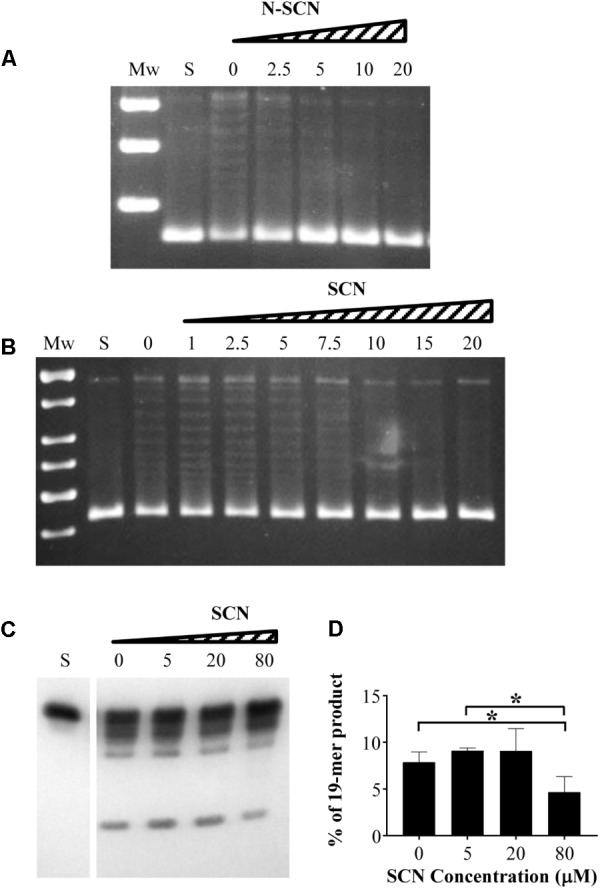
Inhibition of *M. tuberculosis* topoisomerase I by SCN and *N*-SCN. **(A,B)** Supercoiled pBR322 (0.5 μg) was treated with 1 unit of purified MtbTopoI in 200 μl reactions containing *N*-SCN, SCN, or boldine at the concentrations (μM) indicated. Mw, molecular mass standard; S, covalently closed supercoiled pBR322 used as substrate. **(C)** One unit of MtbTopoI was pre-incubated with SCN at the indicated concentrations, then 0.1 pmol of 5′-biotin-labeled 32-mer oligonucleotide was added and the reaction was continued for 30 min. The products were resolved in 12% denaturing PAGE. **(D)** Quantification of the 19-mer cleavage product. The values (mean ± SD) of three independent experiments are shown. ^∗^ indicates statistical significance by Student *T*-test *P <* 0.05.

We have previously reported inhibition of growth and inhibition of *S. pneumoniae* TopoI by 18 semisynthetic compounds derived from the natural alkaloid boldine (García et al., 2011). Out of these, two phenanthrene alkaloids, *N*-SCN and SCN, showed the greatest inhibition of *S. pneumoniae* growth. These were selected to be assayed for inhibition of MtbTopoI. While boldine did not inhibit the enzyme, even at concentrations up to 500 μM (data not shown), MtbTopoI relaxation activity was inhibited in a concentration-dependent manner with *N*-SCN (**Figure 2A**), with IC_50_ values (average ± SD) of 5.6 ± 0.8 μM (*n* = 3). The inhibition by SCN was comparable, with IC_50_ values of 8.4 ± 0.3 μM (*n* = 3) (**Figure 2B**). A good correlation between the inhibition of MtbTopoI activity and the inhibition of cell growth was observed for these compounds. Additionally, inhibition of cleavage of a 32-mer single-stranded DNA with SCN was detected (**Figure 2C**). Significant decreases (*P* < 0.05) of 1.7- and 1.9-fold (**Figure 2D**) in the amount of the 19-mer product were observed at 80 μM SCN when compared with the untreated sample and 5 μM treated samples, respectively. These results imply that MtbTopoI is indeed the *in vivo* target of SCN and *N*-SCN.

### Targeting of *M. tuberculosis* TopoI *in Vivo* With *N*-SCN

**FIGURE 3:**
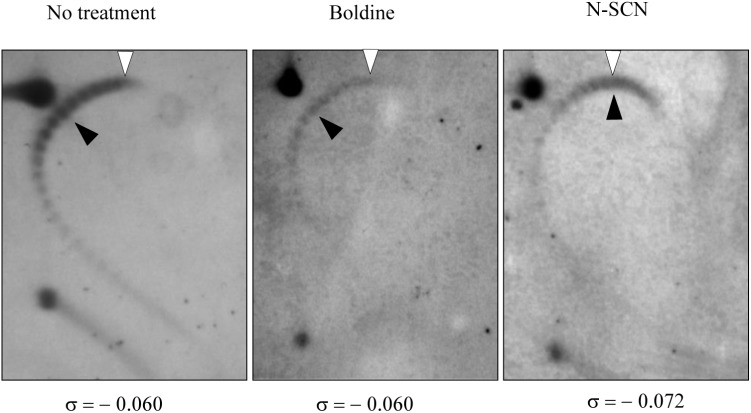
Treatment with *N*-SCN causes hypernegative supercoiling in plasmid DNA. An exponentially growing culture of the *M. tuberculosis* H37Rv strain carrying plasmid pSUM36 was treated at OD_600_ = 0.4 with *N*-SCN or boldine at 1 × MIC. Purified plasmid DNA from samples collected at 24 h after treatment was subjected to two-dimensional agarose gel electrophoresis in the presence of 5 μg/ml chloroquine in the first dimension and 15 μg/ml in the second dimension. An empty arrowhead indicates the topoisomer that migrated with ΔLk = 0 in the second dimension. A black arrowhead indicates the most abundant topoisomer. The corresponding supercoiling density (σ) is indicated below each autoradiogram.

Evidence of the inhibition *in vivo* of MtbTopoI by *N*-SCN was obtained from the analysis of topoisomer distribution of the mycobacterial plasmid vector pSUM36 by two-dimensional agarose gel electrophoresis, a suitable approach for studying supercoiling levels (Ferrándiz et al., 2010). Treatment of pSUM36-containing *M. tuberculosis* cultures with *N*-SCN at 1 × MIC resulted in a 20% increase of plasmid supercoiling (**Figure 3**). Supercoiling density (σ) was -0.060 in the non-treated sample and in 1 × MIC boldine-treated sample, and -0.072 in 1 × MIC *N*-SCN-treated sample, indicating that plasmids became hypernegatively supercoiled. Although direct extrapolation from observations made in small plasmids is not completely equivalent to the bacterial chromosome, our results indicate that supercoiling significantly increases in the presence of *N*-SCN, and support the model that MtbTopoI is its target *in vivo*.

### Elucidation of the Mechanism of Action of SCN and *N*-SCN in Mycobacteria

*Mycobacterium smegmatis* was used as a model for determining the mechanism of action of SCN and *N*-SCN in mycobacteria. The MsPptrtopoI strain of *M. smegmatis* is a conditional knock-down strain which normally produces 1.5 less MsTopoI enzyme than wild type cells, and this level can be reduced further by 2.5 times in the presence of ATc (Ahmed et al., 2015). We observed that, accordingly to the reduction in the levels of MsTopoI enzyme, the MIC of SCN and *N*-SCN against *M. smegmatis* strains decreased (**Table 2**), whereas in contrast, the MIC of imipramine (a well-known topoisomerase poison; Godbole et al., 2015) increased. These experiments suggested that, since reduced levels of MsTopoI render the bacteria more susceptible to SCN and *N*-SCN, MsTopoI would be the target *in vivo* of these two inhibitors.

**Table 2:** Correlation between MICs of SCN and *N*-SCN and levels of MsTopoI.

			MICs (μM)		
*M. smegmatis* strain	Levels of TopoI	Imipramine	*N*-SCN	SCN	Boldine
mc^2^155	+++	125–200	100	30–60	>300
MsPptrtopoI	++	175–300	50	15–30	>300
MsPptrtopoI + ATc	+	350	25	15	>300

*ATc, anhydrotetracycline was added at a concentration of 200 ng/ml. Topoisomerase I levels in M. smegmatis strains: +++: wild type levels; ++: 1.5 times below wild type levels; +: 2.5 times below MsPptrtopoI levels. Estimations according to Ahmed et al. (2015).*

**FIGURE 4:**
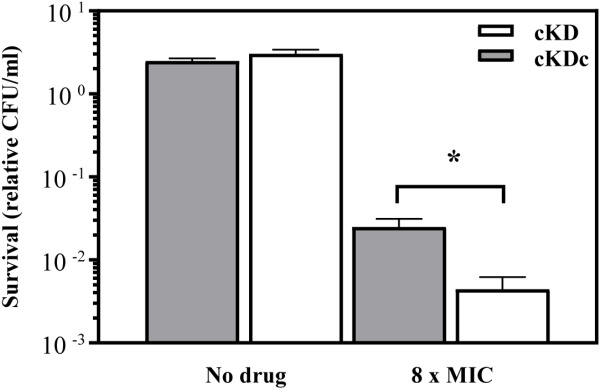
Killing activity of inhibitory concentrations of *N*-SCN on *M. smegmatis*. After a 24-h treatment with an inhibitory concentration of *N*-SCN (8 × MIC), the complemented knock-down strain MsPptrtopoI strain (gray bars) was killed at a lower rate than its non-complemented parental strain (white bars); this difference was significant (*P* < 0.05). The mean ± standard error of the mean from at least three independent replicates is shown.

In order to corroborate this finding, the kill time kinetics of inhibitory concentrations of *N*-SCN on *M. smegmatis* strains was determined after time. We compared the survival of the conditional knock-down mutant of *M. smegmatis* (MsPptrtopoI strain) with that of its corresponding complemented strain, which harbored a plasmid-encoded copy of the *M. smegmatis* *topA* gene. The complemented strain survived better than the parental one in the presence of 8-times the MIC of *N*-SCN, which can be attributed to a higher content of MsTopoI enzyme in the complemented strain (**Figure 4**).

## Discussion

The use of antimicrobials is one of the most effective ways to combat infectious diseases. In the case of TB, effective drugs are scarce: these are just four first-line drugs used in the standard treatment of TB caused by drug susceptible strains. Meanwhile, the incidence of drug resistant TB is increasing, despite the fact that the overall incidence of TB is decreasing slightly. This leaves a very limited number of therapeutic options. This situation has prompted investigation into new drugs, new targets, and new therapies for curing TB (Zumla et al., 2013, 2014). Natural products have been an extraordinary source of antimicrobials in general (Newman and Cragg, 2012; Cragg and Newman, 2013) and of anti-TB compounds in particular (Salomon and Schmidt, 2012). Natural products frequently lack some of the pharmacological properties desirable for a drug, making it necessary to develop semisynthetic products by chemical modifications. Boldine is a natural alkaloid produced by the tree *Peumus boldus* and has no significant antimicrobial activity. We have reported that two boldine derivatives (SCN and *N*-SCN) showed significant anti-pneumococcal activity (García et al., 2011). In this study, we have characterized the anti-mycobacterial activity of these drugs, along with the inhibitory activity on their potential target, the topoisomerase I enzyme.

*Mycobacterium tuberculosis* strains were susceptible to low concentrations of SCN or *N*-SCN. The reference strain H37Rv was the least susceptible strain, but even here the MIC was 15.6 μM (equivalent to 5 μg/ml). Other clinical isolates were susceptible at concentrations of 1.95 μM (equivalent to 0.65 μg/ml). Although these values are greater than the MICs of the most potent anti-TB drugs, isoniazid and rifampicin, they are in the same league as the MICs of ethambutol (another first-line antituberculosis drug) for strains susceptible to this drug (Christianson et al., 2014). The variation in the MICs among the various *M. tuberculosis* clinical strains could be related to their repertoire of nucleoid-associated proteins. For example, it has been shown that one of these proteins, HU, specifically regulates MtbTopoI activity (Ghosh et al., 2015). In any case, the range of MICs observed with SCN and *N*-SCN coincides with the IC_50_ values of the MtbTopoI enzyme (ca. 8 μM). This suggests that both SCN and *N*-SCN can efficiently cross the mycobacterial cell wall, which is a highly impermeable barrier that in general limits the *in vivo* activity of inhibitors shown to be potent enzyme inhibitors *in vitro*.

The comparison of MICs for *S. pneumoniae* (García et al., 2011) and *M. tuberculosis* showed that the latter is more susceptible to the Topo I inhibitors SCN and *N*-SCN. This fact could be related to the differences in the set of DNA topoisomerases available to each species. Both species have a single type I enzyme, but while *S. pneumoniae* possesses two type II enzymes (gyrase and topoisomerase IV), *M. tuberculosis* lacks topoisomerase IV (which carries out efficient DNA relaxation in addition to decatenation). This leaves gyrase as the only type II enzyme in *M. tuberculosis* (Manjunatha et al., 2002; Kumar et al., 2012). Altogether, this enzyme complement must maintain the appropriate level of chromosome supercoiling; in *M. tuberculosis*, gyrase carries out the dual function of both negative supercoiling and decatenation (Cole et al., 1998). Therefore, in the absence of topoisomerase IV, the only mycobacterial enzyme responsible for DNA relaxation is Topo I. Consequently, the inhibition of this enzyme by SCN and *N*-SCN would be more lethal in *M. tuberculosis* than in *S. pneumoniae*. Therefore, it would be worthwhile to investigate the antimicrobial activity of SCN and *N*-SCN in other bacterial pathogens, which also lack topoisomerase IV, such as *Helicobacter pylori* (Debowski et al., 2012).

The topoisomer distribution of the plasmid pSUM36 under control conditions showed a mean superhelical density of -0.060, a figure compatible with that reported for *E. coli* (Deng et al., 2005) and *S. pneumoniae* (Ferrándiz et al., 2010, 2016). Under treatment with *N*-SCN at 1 × MIC, a modest increase (20%) in pSUM36 superhelical density was observed in *M. tuberculosis*. This value is lower than the >58% observed due to treatment of *S. pneumoniae* with SCN at 1 × MIC, but close to the ±20% variation displayed during normal *E. coli* cell growth (Drlica, 1992). These results may indicate that, given the scarcity of the DNA topoisomerase complement of *M. tuberculosis*, the capacity of this bacterium to tolerate changes in supercoiling levels is more limited than with *E. coli* or *S. pneumoniae.*

The investigation of MtbTopoI as a drug target began very recently; when its requirement for cell survival was demonstrated (Ahmed et al., 2014). Until now, several MtbTopoI inhibitors have been discovered. The small molecule amsacrine, a eukaryotic type II topoisomerase poison used as an anti-neoplastic, is an inhibitor of this enzyme in *M. tuberculosis* (Godbole et al., 2014). Imipramine and norclomipramine are two drugs used clinically as antidepressants that also inhibit MtTopoI (Godbole et al., 2015). All these compounds are considered as topoisomerase poisons, since they perturb the cleavage–religation equilibrium and result in the accumulation of enzyme–DNA covalent adducts, which ultimately kill the bacterial cell (Nagaraja et al., 2017). There are other classes of compounds that directly inhibit TopoI (Sandhaus et al., 2016, 2018). SCN and *N*-SCN form part of this last class of compounds. Mechanistically, both types of inhibitors can be distinguished because increases in the levels of TopoI lead to a decrease in the MIC in the case of topoisomerase poisons, or to an increase in the MIC in the case of topoisomerase inhibitors (Nagaraja et al., 2017). In the case of SCN and *N*-SCN, the results we have obtained in this study using mycobacterial strains and in our previously published study with *S. pneumoniae* (García et al., 2011) indicate that they belong to the topoisomerase inhibitors class, not to topoisomerase poisons, since in both microorganisms we detected that increases in the quantity of TopoI corresponded with increases of their MICs. In fact, we showed that the inhibitory effect exerted by these alkaloids was enhanced prior to DNA binding, and in consequence, avoidance of DNA binding resulted in strong inhibitory effects (García et al., 2011).

Concerning toxicity, however, given the potent activity of amsacrine on mammalian TopoII, which results in cell death, it is unlikely that this compound could be used clinically against *M. tuberculosis* without further chemical modifications. We have reported that SCN and *N*-SCN only partially inhibited human TopoI at concentrations of 50 μM, well above the inhibitory concentrations we report in this work for both the enzyme and the bacterial cultures (García et al., 2011). In addition, SCN and *N*-SCN did not cause apoptosis of neutrophil even at 100 μM and only slightly affected neutrophil survival at this concentration (García et al., 2011). In summary, the low toxicity of these compounds makes them very attractive leads for further anti-TB drug development.

## Author Contributions

MG, DC, and JT-V performed the experiments related with cloning of the MtbTopoI gene in *E. coli*, protein expression, purification, and inhibitory assays *in vitro*. MF carried out the analysis of plasmid supercoiling. MA carried out the cleavage assay. LR and BG carried out the experiments with mycobacteria. JA and AC conceived, designed, supervised the study, and wrote the manuscript. All authors actively participated in the correction of the manuscript. The manuscript has been approved by all authors for publication.

## Conflict of Interest Statement

The authors declare that the research was conducted in the absence of any commercial or financial relationships that could be construed as a potential conflict of interest.
